# A serious adverse drug reaction probably induced by clonazepam: a case report of myotoxicity

**DOI:** 10.1186/s40360-019-0366-y

**Published:** 2019-11-05

**Authors:** Xiaonian Han, Jinping Wang

**Affiliations:** Department of Pharmacy, Xi’an Central Hospital, Houzaimen No.185, North Street, Xi’an, 710003 Shaanxi China

**Keywords:** Myotoxicity, Creatine phosphokinase, Clonazepam, Polio

## Abstract

**Background:**

The adverse drug reactions (ADRs) related to clonazepam are mild, and only two cases of myotoxicity induced by clonazepam have been reported, with both patients recovering well. We present a unique case of a serious ADR outcome after taking clonazepam.

**Case presentation:**

A 24-year-old woman with a long-standing history of polio and a 2-year history of epilepsy developed a serious ADR after repeated exposure to oral clonazepam combined with sodium valproate that manifested as myotoxicity and elevated levels of creatine phosphokinase. The patient is currently bedridden and unable to take care of herself.

**Conclusion:**

Clinicians should be vigilant of the possibility of myotoxicity induced by clonazepam, especially in specific populations such as polio patients or when clonazepam is used in combination therapies.

## Background

Epilepsy is one of the most common serious disorders of the brain, affecting about 50 million people worldwide. Nearly a quarter of these patients have drug-refractory epilepsy [1]. Clonazepam is one of the 1,4-benzodiazepine drugs commonly used in epilepsy management, and it is recommended as a second-line adjunctive treatment for various types of seizure [2]. Studies have shown that clonazepam should be used primarily as an adjunctive therapy in patients with different types of drug-resistant primarily and secondarily generalized seizures [3]. The main adverse events are depression, somnolence, dizziness, nervousness, ataxia and reduced intellectual ability [4], and reports of myotoxicity induced by clonazepam are rare. Gupta [5] reported a 15-year-old boy who developed myotoxicity and elevated levels of creatine phosphokinase (CPK) after three doses of oral clonazepam, while Chen [6] reported another 63-year-old woman who suffered from mitochondrial myopathy after the long-term oral administration of clonazepam. All symptoms of these two patients gradually resolved, with the CPK level decreasing to the normal reference range after drug withdrawal.

There has been no previous report on serious adverse drug reactions (ADR) associated with clonazepam. Here we describe a patient who developed myotoxicity and became bedridden after repeated exposure to clonazepam combined with sodium valproate.

## Case presentation

A 24-year-old woman had experienced non-progressive polio from 1 year of age despite having been inoculated with polio vaccine on time. She could walk with a limp but could not control her right hand sufficiently well to write, and so had not received the usual schooling. However, she could take care of herself and help her parents with a small amount of housework. About 2 years previously she had suffered from epilepsy in the form of bilateral tonic-clonic seizures, but she had not received regular treatment due to financial reasons. The epilepsy had not resulted in any deterioration of her polio symptoms.

The patient had been taken to the hospital to receive regular treatment for epilepsy for the first time 6 months previously, at which time her antiepileptic regimen was sustained-release sodium valproate (500 mg p.o.) plus clonazepam (2 mg p.o.) every 12 h. The patient developed drowsiness after the first combined dose of sodium valproate and clonazepam, and slept from 3 p.m. on the first day to 10 a.m. on the following day. She could not walk unaided and felt very tired, but she did not contact her doctor, instead continuing on this antiepileptic regimen regularly for 21 days until she ran out of clonazepam tablets. Her epilepsy symptoms were well controlled during this 21-day treatment period, but she developed muscle weakness and muscle pain, and remained in bed since she could not take care of herself.

The patient continued taking the sustained-release sodium valproate tablets (500 mg p.o. every 12 h), during which her epilepsy symptoms remained well controlled. Her muscle weakness symptoms started to improve gradually over the following 2 weeks, allowing her to stand but not walk. At this time she was taken to hospital for the second time. The patient and her family unfortunately refused electromyography, a muscle biopsy and other tests with the exception of some simple blood tests due to financial reasons. The findings of preliminary blood examinations including the complete blood count, urine test, liver and kidney function test, electrolytes, and plasma ammonia were within clinically acceptable limits, but her serum CPK was markedly raised at 4261 U/L (38–174 U/L), while her serum sodium valproate concentration was 101.89 μg/mL (50–100 μg/mL) and her serum globulin concentration was 36.4 g/L (25–35 g/L). There had been no preceding illness, infection or trauma, the patient did not have a history of statins or other drugs, and there was no family history of any neuromuscular disorder. Based on the relationship between medication times and symptoms, we attributed the myotoxicity to clonazepam, and assigned the patient to an antiepileptic regimen of sodium valproate monotherapy, with clonazepam remaining discontinued.

A 2-month telephone-based follow-up revealed that the patient had started taking clonazepam irregularly because of insomnia, and suffered from muscle weakness and muscle pain again. Her clinical condition had deteriorated to the point that she was unable to stand or walk, and was unable to take care of herself. The patient and her family refused further physical examinations and treatment because they had lost confidence in curing the disease and also for financial reasons. We recommended that the patient discontinued clonazepam immediately and never take it again. At another follow-up 3 months later, the frequency and severity of epileptic seizures were significantly reduced in the patient, but her myotoxicity condition had not improved, she still could not stand or walk, and she was now bedridden.

## Discussion and conclusion

Our patient developed muscle weakness after taking sodium valproate and clonazepam at the same time, and the myopathy caused by sodium valproate and by clonazepam had been reported [5–9]. However, the clinical symptoms in the present patient gradually improved when clonazepam was stopped while continuing sodium valproate, and the patient experienced muscle weakness again after restarting clonazepam. There had been no preceding known illness, infection or trauma, the patient denied taking any other medications or substances, and there was no family history of any neuromuscular disorder. Although the patient had a long-standing history of polio and a 2-year history of epilepsy, these conditions had been non-progressive. We decided to substantiate the diagnosis further by using the Naranjo ADR Probability Scale, which is used to determine the likelihood of an ADR being due to the implicated drug or other factors [10] (Table 1). Our patient scored 6 on this scale, which indicates a probable ADR, and so her myotoxicity was probably related to the taking of clonazepam.

**Table 1:** Naranjo adverse drug reaction probability score

No.	Item	Yes	No	Do not know	Score
1	Are there previous conclusive reports of this reaction?	+ 1	0	0	+ 1
2	Did the adverse reaction event appear after the suspected drug was administered?	+ 2	−1	0	+ 2
3	Did the adverse reaction improve when the drug was discontinued or a specific antagonist was administered?	+ 1	0	0	+ 1
4	Did the adverse reaction reappear when the drug was readministered?	+ 2	−1	0	+ 2
5	Are there alternative cause (other than the drug) that could on their own have caused the reaction?	−1	+ 2	0	−1
6	Did the reaction reappear when a placebo was given?	−1	+ 1	0	0
7	Was the drug detected in the blood (or other fluids) in concentrations knows to be toxic?	+ 1	0	0	0
8	Was the reaction more severe when the dose was increased or less severe when the dose was decreased?	+ 1	0	0	0
9	Did the patient have a similar reaction to the same or similar drugs at any previous exposure?	+ 1	0	0	0
10	Was the adverse event confimed by any objective evidence?	+ 1	0	0	+ 1
				Total	6

Definite:≥9; Probable: 5–8; Possible: 1–4; Doubtful: ≤0

The patient and her family declared that her polio and epilepsy were non-progressive. However, the dearth of neurological examinations meant that the woman might have actually had a slowly progressive early-onset type of muscle-wasting disease of neurological or myopathic origin, whose acceleration by the epilepsy treatment could have caused her clinical manifestations. Moreover, the probable myopathy varied with her clonazepam intake, representing further evidence that clonazepam was related to the myotoxicity. Numerous agents exhibit well-documented myocytoxicity, including anticholesterol statins, antirheumatic/inflammatory/immunosuppressive drugs, antinucleoside analogues, contaminated products, and dietary agents, and they result in symptomatologies ranging from mild discomfort and inconvenience to permanent damage and disability [11, 12]. The toxic myopathies can be classified according to the type of injury induced in the muscle fibre or specific organelle as follows: necrotizing myopathy, inflammatory myopathy, thick-filament loss myopathy, type II fibre atrophy, mitochondrial myopathies, lysosomal storage myopathies, antimicrotubular myopathies, myofibrillar myopathies, and fasciitis [11]. Cases of myotoxicity induced by clonazepam have probably been classified as mitochondrial myopathies, as confirmed by COX, NADH and SDH staining of the muscle biopsy samples of a 63-year-old woman [6]. Performing a muscle biopsy is essential to document myotoxicity, but regrettably we were only able to perform blood and urine tests in our patient, and so the lack of EMG and muscle biopsy results represent limitations of this study. We could therefore only speculate that the myotoxicity in our patient was mitochondrial myopathy.

Because our patient was taking sodium valproate at the same time as clonazepam, drug interactions might have been present. Jeavons et al. [13] reported the development of absence status in 5 of 11 patients taking both sodium valproate and clonazepam. Sodium valproate is an enzyme inhibitor and clonazepam is metabolized by cytochrome P450 in vivo [14, 15], which could explain why the patient experienced prolonged sedation. Baruzzi et al. [16] measured the serum levels of sodium valproate and clonazepam in epileptic patients treated with multiple antiepileptic drugs, and found only a weak correlation between the dose of clonazepam and its plasma level, which contrasts with other reports on clonazepam as a monotherapy [17, 18]. This observation was attributed to drug interactions, and so a pharmacodynamic drug interaction between sodium valproate and clonazepam cannot be completely excluded as a cause of an ADR in the present patient.

The reported myocytoxicity symptoms including ataxia, fatigue and weakness induced by clonazepam were resolved and the CPK level reduced to the normal reference range after stopping clonazepam in our patient [5, 6]. The disability experienced by our patient might have resulted from immobility and prolonged sedation, or an interaction between sodium valproate and clonazepam, but it could also have been related to the history of polio, despite the lack of evidence for clonazepam being forbidden in polio. Furthermore, the recognition of ADR and the lack of medical knowledge were also contributing factors to this serious ADR. It was a great pity that our patient refused to receive further physical examinations and treatment due to losing confidence in curing the disease and also for financial reasons.

We have described a case of the serious ADR of myotoxicity that was probably induced by clonazepam. Our findings suggest that clinicians should be vigilant of the possibility of myotoxicity induced by clonazepam, especially in specific populations such as polio patients or when clonazepam is used in combination therapies.

## Data Availability

The data used in this case report is available from the corresponding author on reasonable request.

## Abbreviations

ADR: Adverse drug reaction
AZT: Azidothymidine
CPK: Creatine phosphokinase
EMG: Electromyogram
